# Intraperitoneal Instillation of Lidocaine for Postoperative Pain Relief after Total Abdominal Hysterectomy: A Double Blinded Randomized Placebo-controlled Trial

**DOI:** 10.22037/ijpr.2020.1101084

**Published:** 2020

**Authors:** Ziba Zahiri Sorouri, Forozan Milani, Abtin Heidarzadeh, Masoumeh Akhavan Azari

**Affiliations:** a *Reproductive Health Research Center, Department of Obstetrics and Gynecology, Alzahra Hospital, School of Medicine, Guilan University of Medical Sciences, Rasht, Iran. *; b *Vice-Chancellorship of Research and Technology, Guilan University of Medical Science, Rasht, Iran.*

**Keywords:** Hysterectomy, Pain, Lidocaine, Intraperitoneal

## Abstract

Pain after total abdominal hysterectomy (TAH) is a major concern. Pain management is very important issue after TAH. This study aimed to assess the efficacy of intraperitoneal instillation of lidocaine for postoperative pain relief after TAH. A double-blinded randomized placebo-controlled trial was conducted on patients undergoing total abdominal hysterectomy in Al-zahra hospital from June 2007 to July 2008. Forty patients were randomly assigned with equal number in two lidicaine (N = 20) and normal saline (N = 20) groups. The lidocaine group received 50 mL of 0.8% lidocaine with epinephrine and placebo group received 50 ml of saline 0.9%. We used 10 cm visual analog scale (VAS) for assessing pain at 8, 12, and 24 h at rest and 48 h on movement. Opioid consumption, patient’ satisfaction with pain control, and incidence of postoperative nausea and vomiting were assessed. Means of pain score at different times in lidocaine group were significantly lower than placebo group (*P *˂ 0.05) the difference between mean dose of opioid consumption over 24 h between two groups was not significant (*P *= 0.785). Patient’s satisfaction score in lidocaine group was significantly higher than saline group *(P *= 0.034). Differences in incidence of postoperative nausea and vomiting between two groups were not significant (*P *= 1.0). Intraperitoneal instillation of 50 mL of 0.8% lidocaine with epinephrine is an effective and safe technique for postoperative pain management after TAH. But this technique cannot reduce opioid consumption over 24 h after TAH.

## Introduction

Abdominal hysterectomy is a prevalent surgery in women and causes extensive tissue injury. Pain after total abdominal hysterectomy (TAH) is a major concern and induces physical and psychological complications that can increase the length of the hospitalization (1, 2). Pain is usually classified into two main categories by the type of damage that causes it. These two categories are nociceptive and neuropathic pains. During surgery, tissue injury causes nociceptive pain and nerve damage causes neuropathic pain (1). Postoperative pain relief is very important issue in any surgery. The common postoperative pain management is traditionally based on opioids (4, 5). Considering opioids’ adverse effects such as nausea, vomiting, sedation, drowsiness, and urinary retention, recently there is a lot of interest for finding a safe and effective pain treatment after operation. Several new methods are introduced for postoperative pain relief (5-7). But there is not enough evidence about clinically impact of these techniques (8). Postoperative pain relief provides patient comfort and allows the patient to breathe, cough and move sooner. Also, it can reduce the incidence of complications after surgery (5, 9 and 10).

Local anesthetics are recognized as a useful technique for postoperative pain management. They can reduce inflammatory response after surgery and produce analgesia by blocking neural transmission at the site of tissue injury (9). A suitable local anesthetic should be effective, safe, and inexpensive. Lidocaine is a proper and the most widely used local anesthetic. Lidocaine is used in several ways for managing the postoperative pain. Clinical studies that examine efficacy of lidocaine for postoperative pain relief, reported mixed results. Reduction in postoperative pain and opioids consumption are shown in several studies, whereas some studies failed to demonstrate beneficial effects (10-20). In this study we aimed to assess the efficacy of intraperitoneal instillation of lidocainefor postoperative pain relief after TAH.

## Experimental

*Trial design and setting*

A two-arm parallel double-blinded randomized placebo-controlled trial with equal randomization (1:1) was conducted on patients undergoing total abdominal hysterectomy in Al-zahra hospital from June 2007 to July 2008. This hospital is the only governmental maternity hospital in Rasht in Guilan province, north of in Iran.

*Patients*

Eligible patients were all women who were undergoing total abdominal hysterectomy. Patients were excluded if they had history of hepatic, cardiovascular diseases, asthma, seizure disorder, cancer, chronic pain, opioid addiction, hypersensitivity or allergy to local anesthetics, or require further surgeries.

This study was approved by the Ethical Committee of Guilan University of Medical Sciences. All patients provided written informed consent before inclusion in the study.

*Randomization and Interventions*

A co-worker assigned participants to lidocaine and placebo groups using a random sequence that was prepared by a statistician with no clinical involvement in the trial. The randomization list was generated using permuted block size of four. Sealed envelopes labeled A or B were provided. After assignment, one of them was delivered to operating room. We put in each envelope one Syringe containing lidocaine or normal saline. Syringe for the lidocaine group containing 50 mL of 0.8% lidocaine with epinephrine (1:500000 dilution, prepared by adding 30 mL of normal saline to 20 mL of 2% lidocaine containing epinephrine 1:200000) and in the placebo group syringe containing 50 mL of normal saline 0.9%. All patients received a standardized general anesthesia were induced with intravenous injection of sodium thiopental 4 mg/kg, fentanyl 2 µg/kg and neuromuscular blockade by 0.5 mg/kg atracurium. Tracheal tube was inserted and anesthesia was maintained by isoflurane, nitrous oxide and oxygen. Atracurium was repeated as necessary. The hysterectomy was performed using standard technique; Surgery in all patients was performed via a standard Pfannenstiel incision after closureof vaginal cuff and visceral peritoneal, content of syringes was administered into the peritoneal cavity. At the end of surgery, neuromuscular blockade was reversed by administration of atropine 0.02 mg/kg and neostigmine 0.04 mg/kg and the patients were extubated. In this trail, patients, research personnel, outcome assessor, and ward nursing staff were blind of group allocation.

*Data collection and outcomes*

Primary outcome of this trial was pain scores at 8, 12, and 24 h at rest and 48 h on movement (induced by sitting). We used 10 cm visual analog scale (VAS) (0 = no pain; 10 = worst pain) for assessing pain. Before surgery all participants were educated about use of VAS. Also we collected data about duration of surgery, Opioid consumption over 24 h after surgery, and incidence of postoperative nausea and vomiting. Also patients’ satisfaction with pain control was assessed by interviewing at 24 h after surgery using a scale of 1 = poor, 2 = moderate, and 3 = good. Preoperative data including age, body mass index (BMI), and history of abdominal surgery were obtained by nurses that were no clinically involved in the trial.

*Sample size*

To detect a difference in 0.9 cm in pain score between two groups with an error probability of 5% and a power of 80%, assuming a standard deviation (SD) of 1 cm, a sample size of 20 patients per group was needed.

*Statistical analysis*

Data were analyzed in IBM SPSS Software Version 21. Descriptive and analytic statistics were used. Numeric data were shown as mean and SD and categorical variables data were shown as number and percentage. For statistical analysis, the non-parametric Mann Whitney U test was used to compare pain scores in different postoperative times and satisfaction scores between two groups. Two-tiled independent *t*-test was used to compare mean dose of opioid consumption, duration of surgery, and age between two groups. Also Fisher’s exact tests were used to compare proportions between two groups. A *P*-value less than 0.05 has been considered as statistically significant.

## Results

**Figure 1:**
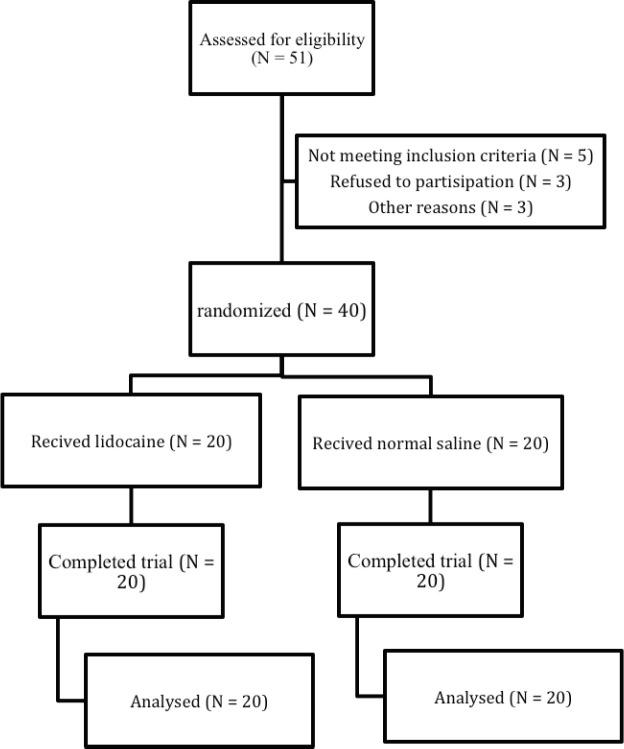
Flow of participants

During the period of study 51 patients underwent total abdominal hysterectomy were assessed for eligibility. Forty patients were randomly assigned with equal number in two lidicaine (N = 20) and normal saline (N = 20) groups. All participants completed the trial (Figure 1). Baseline characteristics of the participants in two groups are shown in Table 1.

**Figure 2.**
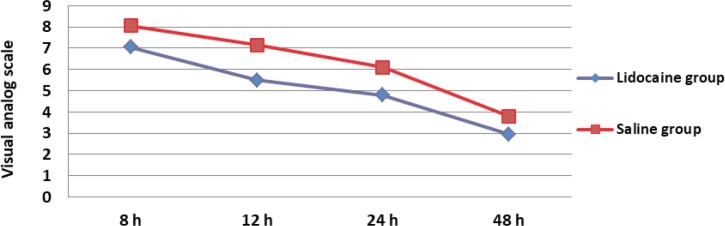
Pain scores after total abdominal hysterectomy using a visual analog scale in two lidocaine and saline groups

Mean of duration of surgery in lidocaine group was 63 ± 13.41 min and in saline group was 66 ± 18.47 min that this difference was not significant (P = 0.560). Mean dose of pethidine consumption over 24 h after surgery in lidocaine group was 106.25 ± 29.10 mg and saline group was 108.75 ± 28.42 mg that this difference between two groups was not significant (P = 0.785). Means of pain score at different time in lidocaine group were significantly lower than normal saline group (P ˂ 0.05) (Table 2, Figure 2). In lidocaine group 9 patients (45%) and in normal saline group 3 patients (15%) defined their satisfactions as good. Mean of patient’s satisfaction score in lidocaine group was 2.20 ± 0.61 and in normal saline group was 1.5 ± 0.51 that this difference between two groups was statistically significant (P = 0.034). Five patients (25%) in lidocaine group and 5 patients (25%) in normal saline group experienced nausea, also vomiting by 4 (20%) in lidocaine group and 5 (25%) in saline group was experienced, that these differences between two groups were not significant (P = 1.0, P = 1.0).

## Discussion

Lidocaine is the most widely used local anesthetic. Lidocaine like other sodium channel blockers reduces pain by neural impulses blocking (21). To prolong the postoperative pain relief effect of lidocaine, and reducing hepatic exposure and the risk of systemic reaction, lidocaine is usually administrated in combination with vasoconstrictor such as epinephrine (15, 22).

 The results of this study demonstrate that administration of intraperitoneal lidocaine with epinephrine during TAH can significantly reduce postoperative pain at 8, 12, and 24 h at rest and 48 h on movement compared with normal saline. Also patients in lidocaine group were significantly more satisfied with pain control than normal saline group. But this technique cannot reduce pethidine consumption over 24 h after TAH. The incidence of nausea and vomiting in both groups was similar.

Consistent with our findings in Williamson et al. study that examined intraperitoneal lignocaine instillation for pain control after TAH, pain score at 24 h at rest was lower in lignocaine group compared with saline significantly. Also there was no significant difference in morphine consumption between two groups. But in contrast with our finding there was no significant difference in pain score at 48 h on movement between two groups (15). Difference between our finding and this study in 48 h on movement may be due to the concentration of lidocaine, we used 50 mL of 0.8% lidocaine but in Williamson et al study 50 mL of 0.4% lidocaine was used. In other randomized controlled trial, women who received preemptive analgesia with 20mL of 1% lidocaine at the abdominal incision site prior to the performance of the hysterectomy compared with saline, perceived lower pain at 2, 5 and 8 h significantly (14). Other study that examined efficacy of intraperitoneal lidocaine instillation for post cesarean pain relief showed that pain scores in women who received lidocaine were significantly lower on the first postoperative day (23). Abdelazim et al. in a study that was carried out on patients who were undergoing gynecological laparoscopy found that Intraperitoneal lidocaine can significantly reduce pain and opioid consumption over 24 h after surgery compared with saline (16). Some studies demonstrated efficacy of Intraperitoneal instillation of lidocaine for pain relief after gynecological surgeries (11, 12, 22, 24 and 25). Also studies conducted on patients who were undergoing other abdominal surgeries, demonstrated efficacy of intraperitoneal lidocaine for reducing pain after surgery (13, 26 and 27). A systematic review identified intraperitoneal instillation of local anesthetics during gynecologic laparoscopy significantly decreased pain during a 6 h interval after operation but had no effect at 24 h after that (28). TAH causes extensive tissue injury. Lidocaine is more effective in surgeries that cause more tissue injury because provoke a greater inflammatory response (30), also the rate of absorption of lidocaine is slower after hysterectomy than gynecological laparoscopy (15, 30). Other systematic review including eight randomized trials in gastrointestinal and gynecological surgery intraperitoneal local anesthetic consistent with our findings reduced postoperative pain but not opioid consumption (29).

**Table 1:** Baseline characteristics of participants

***P-*** **value**	**Saline (N = 20)**	**Lidocaine (N = 20)**	**Variable**
0.063	48.20 ± 6.55	47.95 ± 3.44	^*^Age (year)
0.293	28.45 ± 4.56	27.25 ± 2.05	BMI (kg/m^2^)^*^
1.0	8 (40)	9 (45)	^**^History of abdominal surgery

^*^Mean ± SD, ^**^numbers (%).

**Table 2:** Comparison of pain scores at different time after total abdominal hysterectomy between two lidocaine and saline groups

**Time after surgery**	**Pain score in Lidocaine group (N = 20)**	**Pain score in Saline group (N = 20)**	**Mean difference (95% CI)**	***P*** **-value**
8 h	7.05 ± 0.95	8.05 ± 0.89	-1.0 (-1.59, -0.41)	0.002
12 h	5.50 ± 1.0	7.15 ± 1.0	-1.65 (-2.30, -1.0)	0.0001
24 h	4.80 ± 0.89	6.10 ± 1.17	-1.30 (-1.96, -0.64)	0.001
48 h	2.95 ± 1.10	3.80 ± 1.20	-0.85 (-1.59, -0.11)	0.038

## Conclusion

In conclusion, intraperitoneal instillation of 50 mL of 0.8% lidocaine with epinephrine is an effective and safe technique for postoperative pain relief over 24 h at rest and at 48 h on movement after TAH. But this technique cannot reduce opioid consumption over 24 h after TAH.

Further studies that assess pain in shorter time after operation and narrow interval among pain assessment time and assess opioid consumption in each time is recommended.
